# How Not to Face a Consultant

**Published:** 1914-12-05

**Authors:** 


### How Not to Face a Consultant.

From time immemorial the physician has been regarded as the gentleman of medicine in the comprehensive meaning of that word. When surgery began to extend its influence upon the public, to the exclusion of most other methods of treatment, it was felt that the physician might be crowded out. That was a few years ago, but to-day the trend of public feeling is distinctly towards the physician. For it has come to be felt that, like the best of general practitioners, he is before all things the friend of his patient. The moment is therefore ripe to insist that when a man wishes to be overhauled by a physician—and the busier the man is, the more the rule applies—it is essential that he shall make an appointment for an hour before he commences his ordinary avocations. Otherwise he may, as one patient did, find himself at dinner when the physician arrives, have to ascend to his bedroom and submit himself to examination in a condition which was certainly neither just to the physician nor best calculated to promote an accurate diagnosis. It is equally bad to rush from the office into the consulting-room, or to choose a morning for consultation after a long night of hard work, or to motor—or, worse still, to come up on a motor-bicycle some thirty or more miles—to Harley Street for the consultation. These considerations are factors of importance which every man in business should realise and understand. The surgeon necessarily prepares his patient in advance for admission to the operation theatre. And all patients who are wise will prepare themselves for an interview with their physician by endeavouring to be in as normal a condition as possible when each enters his consulting-room.

